# Yellow light improves milk quality, antioxidant capacity, immunity, and reproductive ability in dairy cows by elevating endogenous melatonin

**DOI:** 10.3389/fvets.2025.1730661

**Published:** 2026-01-16

**Authors:** Zixia Shen, Weijia Wang, Xuening Liu, Xiangao Shan, Hao Wu, Guangdong Li, Songyang Yao, Yunjie Liu, Laiqing Yan, Pengyun Ji, Bingyuan Wang, Guoshi Liu

**Affiliations:** 1 National Engineering Laboratory for Animal Breeding, Key Laboratory of Animal Genetics and Breeding of the Ministry of Agricultural, Beijing Key Laboratory for Animal Genetic Improvement, College of Animal Science and Technology, China Agricultural University, Beijing, China; 2 Department of Internal Medicine, Reproduction and Population Medicine, Faculty of Veterinary Medicine, Ghent University, Ghent, Belgium

**Keywords:** antioxidant, cow, immunity, melatonin, production, reproductive, yellow light

## Abstract

**Background:**

Light is an important environmental factor influencing animal production. In livestock production, light management techniques are common. They can enhance production and reproductive performance.

**Aim:**

This study investigated the effects of light wavelength on dairy cows. It focused on production, immunity, antioxidant capacity, and reproduction. It emphasized yellow light.

**Methodology:**

In Experiment 1, 196 cows were divided into three groups and subjected to natural dark, red, and yellow light for 2 weeks. Results indicated yellow light was most effective. This prompted a second experiment. In Experiment 2, 80 postpartum cows received nocturnal yellow light until their next calving. Blood and milk samples were analyzed for immune, antioxidant, and reproductive markers.

**Results:**

The findings demonstrated that yellow light significantly enhanced milk yield (32.39–37.58 kg) and composition, including milk fat percentage, milk protein percentage, lactose percentage, milk urea nitrogen, and somatic cell count. It improved immune status (TNF-α: 181.10–174.90 pg/ml, IL-6: 117.30–113.90 pg ml, IL-10: 31.18–32.86 pg/ml), antioxidant status (superoxide dismutase: 111.80–117.60 U/ml, total antioxidant capacity: 8.28–8.76 U/ml), and superior reproductive performance (the interval to first postpartum estrous cycle: 61.72 ± 1.27 to 56.91 ± 1.14 days, the pregnancy rate after first-insemination: 23.68 ± 4.42% to 38.15 ± 5.00%, the pregnancy days after first-insemination: 96.84 ± 4.88 to 82.95 ± 4.50 days). This was associated with enhanced melatonin levels in serum (36.30–59.48 pg/ml) and milk (20.49–29.22 pg/ml).

**Conclusion:**

Nocturnal yellow light exposure, by elevating endogenous melatonin, is a viable non-invasive strategy to improve overall productivity, health, and welfare in dairy farming.

## 1 Introduction

Dairy productivity is a key determinant of economic profitability on dairy farms. Various environmental factors influence dairy productivity, among which light plays a distinctive role. Natural or artificial light sources exerted broad biological effects on dairy cows, including the regulation of circadian rhythms, physiological activities, metabolism, and behavior (1). In livestock production, light management techniques—through modifications in light wavelength, photoperiod, and intensity—could be employed to modulate animal growth, development, and reproduction. It was demonstrated that UV-B light (peak radiation at 295 nm) during automatic milking increases daily milk yield and vitamin D levels in milk (2). It was found that colostrum yield and IgG content in Jersey cows are negatively correlated with daily light exposure (3). Numerous other studies have also indicated that shortening the photoperiod during the dry period improves milk yield and immunity in dairy cows (4–6). Light intensity influenced milk yield, milk composition, hormone levels, and cortisol concentrations in dairy cows (7). Laser irradiation at 870–970 nm wavelengths was applied to donor cows during superovulation, increasing the number of corpora lutea, recovered embryos, and transferable embryos compared to a non-irradiated control group (8). Another study demonstrated that yellow LED lighting significantly increased dry matter intake, rumination time, and body weight in dairy cows compared to white LED lighting (9). Although research on the effects of light on dairy cows continued, studies focusing on specific light wavelengths remained relatively limited.

Melatonin (N-acetyl-5-methoxytryptamine, MT) is an indoleamine molecule. It was first isolated and identified from bovine pineal glands by Aaron Lerner et al. in 1958 (10). MT is widely distributed among bacteria, fungi, plants, and animals (11). MT exerts a wide range of effects, such as regulating circadian rhythms, providing antioxidant activity, and exerting anti-inflammatory actions (12). In mammals, the synthesis of MT is influenced by the daily photoperiod, wavelength, and intensity of light. Boulanger et al. found that increased MT during periods of prolonged darkness helped protect mammary cells from damage during mammary infections (13). MT levels in milk were also affected by light intensity (7). Compared to blue light, yellow light significantly increased serum MT levels in dairy cows during the night (9). Furthermore, to enhance MT content in milk, many countries had employed light-control management techniques to produce natural high-MT milk, which had been successfully commercialized. Among photoperiod, wavelength, and intensity, light wavelength (also referred to as light color) had the most pronounced effect on MT secretion (14). Therefore, utilizing specific light wavelengths to enhance melatonin levels holds the greatest production significance.

However, existing research on dairy cows had predominantly focused on photoperiod or light intensity. There is a significant gap in the understanding of light wavelength. Thus, this study aims to comprehensively investigate the effects of light wavelength on dairy cows. It focuses on production, immunity, antioxidant capacity, and reproduction, and also tries to elucidate the underlying mechanisms.

## 2 Materials and methods

### 2.1 Chemicals

Methanol was purchased from Thermo Fisher Scientific Co., Ltd. (81 Wyman Street, Waltham, MA, 02454), and anhydrous ethanol was purchased from Sinopharm Group Chemical Reagent Co., Ltd. (Shanghai, China).

### 2.2 Animal study and experimental design

The animal study was approved by the Ethics Committee of the College of Animal Science and Technology, China Agricultural University (AW51704202-1-1). The animal study was conducted at Sanshi Dairy Farm in Changping District, Beijing, China.

#### 2.2.1 Experiment 1

A total of 196 healthy lactating dairy cows (Holstein), with an average body weight of 700 kg, were used in this study. The cows were housed in a semi-open barn (Supplementary Figure 1) and fed four times daily at 06:00, 12:00, 18:00, and 21:00 h, with free access to water. All environmental conditions were kept consistent except for light exposure. The cows were randomly divided into three groups: control group, red light group, and yellow light group. The control group was subjected to a natural photoperiod cycle (no light at night); the red light group received continuous red light (wavelength 620–650 nm, light intensity 350–500 lux) during the natural dark period from 20:30 to 05:30 h the next day; and the yellow light group received continuous yellow light (wavelength 570–600 nm, light intensity 350–500 lux) during the natural dark period from 20:30 to 05:30 h the next day. All lights were controlled by automated timer switches. The study was conducted over a 14-d period. The study lasted for 2 weeks. During the research period, cows were milked daily at 5:00, and milk from the same group of cows was collected in a common large tank. Milk samples from three groups of cows were collected for MT and milk composition analysis.

#### 2.2.2 Experiment 2

Based on the results of the above experiment, we conducted a second phase of the trial: 80 postpartum dairy cows with similar age, body weight, and lactation stage (180 ± 40 days in milk) were randomly selected. They were exposed to continuous yellow light treatment during the natural dark period from 20:30 to 5:30 each day. The 3 days prior to the trial served as the control period (no light treatment). This yellow light management continued until the cows calved again.

Considering the dynamic changes in blood and milk components, prolonged single sampling times could affect the reliability of the results. Therefore, for 35 of these cows, blood and milk samples were collected on the 3 days before the trial (control period), as well as on the 4th, 11th, 18th, and 25th days of the trial. Finally, we tracked the following reproductive performance indicators for these cows: the interval to first postpartum estrous, the pregnancy rate after first-insemination, and the pregnancy days after first-insemination. These results were compared with those of cows that did not undergo light treatment but had similar physiological status to evaluate the effect of yellow light exposure on reproductive performance. Only the authors were aware of the group allocation at the different stages of the experiment.

### 2.3 Collection and processing of milk samples

The milk samples were obtained from each cow at 5:00 on the sampling day and each sample was divided into two tubes. One tube was mixed with potassium dichromate and stored at 4 °C for Dairy Herd Improvement (DHI) test. The other tube was stored at −20 °C for testing the MT content.

### 2.4 Test of DHI indicators in milk

The DHI test was conducted at the Dairy Herd Improvement Testing Station of the Beijing Livestock Station. Fifty microliter of potassium dichromate mixed milk was used for the DHI test. The fat, protein, lactose, and milk UN content in the milk were measured using a MilkoScan FT+ automatic analyzer (Denmark Serial No.9175049, Part No.60027086), and the SCC in the milk was measured using a FossomaticTM FC automatic analyzer (Denmark Serial No. 975377, Part No. 60002326).

### 2.5 Detection of melatonin levels in milk

The milk samples were ultrasonicated for 30 min at 37 °C, then, 1 ml of milk sample was added to 4 ml of methanol, vortexed for 5 min, and then centrifuged 12,000 rpm for 10 min at 4 °C. The supernatant was filtered through a 0.22 μm filter into a brown vial. MT was measured with Agilent 6470 liquid chromatography-tandem MS (LC-MS-MS; Agilent Technologies, Santa Clara, CA, USA).

### 2.6 Collection and processing of blood samples

On the sampling day, before morning feeding (5:30 am), whole blood was collected from the tail vein of cows and transferred into coagulation-promoting tubes. The samples were centrifuged at 3,000 rpm for 10 min at 4 °C, and the serum was then transferred into 5 ml centrifuge tubes and stored at −20 °C.

### 2.7 Test of melatonin levels in serum

Five hundred microliter of serum was mixed with 2 ml of methanol, followed by vigorous shaking in a vortex oscillator for 10 min and then it was allowed to stand at −20 °C for 30 min. Afterward, the supernatant was extracted using a low-temperature high-speed centrifuge 12,000 rpm for 10 min at 4 °C, and filtered through a 0.22 μm filter into a brown vial. MT was measured with Agilent 6470 liquid chromatography-tandem MS (LC-MS-MS; Agilent Technologies, Santa Clara, CA, USA).

### 2.8 Test of antioxidant indicators, reproductive hormones, and immunological parameters

The SOD, MDA, and T-AOC were tested using biochemical methods. LH, E2, and P4 were tested using enzyme-linked immunosorbent assay (**ELISA**). PRL, COR, IL-6, IL-10, and TNF-α were also tested using ELISA. All the testing reagents were procured from Beijing Solarbio Science & Technology Co., Ltd and operated according to the instructions manual.

### 2.9 Statistical analyses

The data were presented as mean ± SEM. If not specifically stated, the difference between two groups was analyzed using *t*-tests, while differences among three or more groups were analyzed using one-way ANOVA followed by the Duncan's multiple range test. Pearson's correlation analysis was employed to determine the degree of association between the two variables. Statistical analysis was performed using GraphPad Prism 8.0.2 software. A value of *P* < 0.05 was considered statistically significant. The confidence interval is 95%.

## 3 Results

### 3.1 Effects of light wavelengths on milk quality and daily milk yield

**Figure 1:**
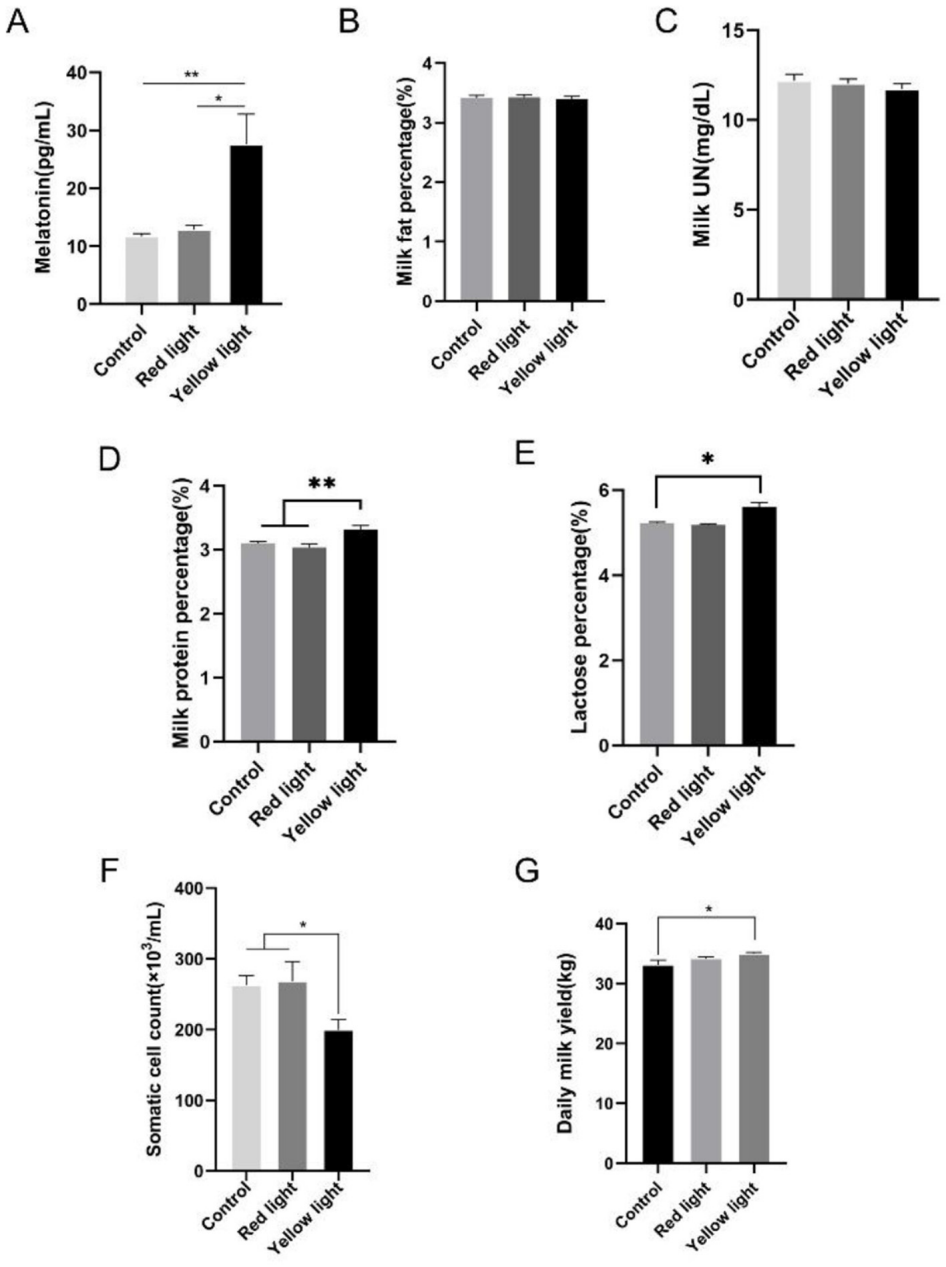
Effects of light management on milk quality and daily milk yield. **(A)** Milk MT content. **(B)** Milk fat percentage, *n* = 29; **(C)** milk urea nitrogen content, *n* = 21; **(D)** milk protein percentage, *n* = 29; **(E)** lactose percentage, *n* = 29; **(F)** somatic cell count of the milk, *n* = 26; and **(G)** daily milk yield, *n* = 18. The data were expressed as mean ± SEM. *P < 0.05, **P < 0.01.

To investigate the effects of different light wavelengths on milk yield and quality, we divided the cows into three groups exposed to darkness, yellow light, or red light at night. The results showed that the MT content in the milk of the cows under yellow light management was significantly higher than in those in the other two groups, while no significant difference was observed between the red light treated cows and the control cows (Figure 1A). There were no significant differences in milk fat percentage and milk UN levels among the groups (Figures 1B, C), but the milk protein percentage in the cows treated with yellow light was significantly higher and the SCC was significantly lower than those in the other two groups (Figures 1D, F). And the lactose percentage in the cows treated with yellow light was significantly higher than the control group (Figure 1E). However, these parameters had no significant differences between the red light and the control group (Figures 1D–F). In addition, the daily milk production of cows with the yellow light illumination was significantly higher than those in the other two groups (Figure 1G).

### 3.2 Effects of yellow light on milk quality and daily milk yield

**Figure 2:**
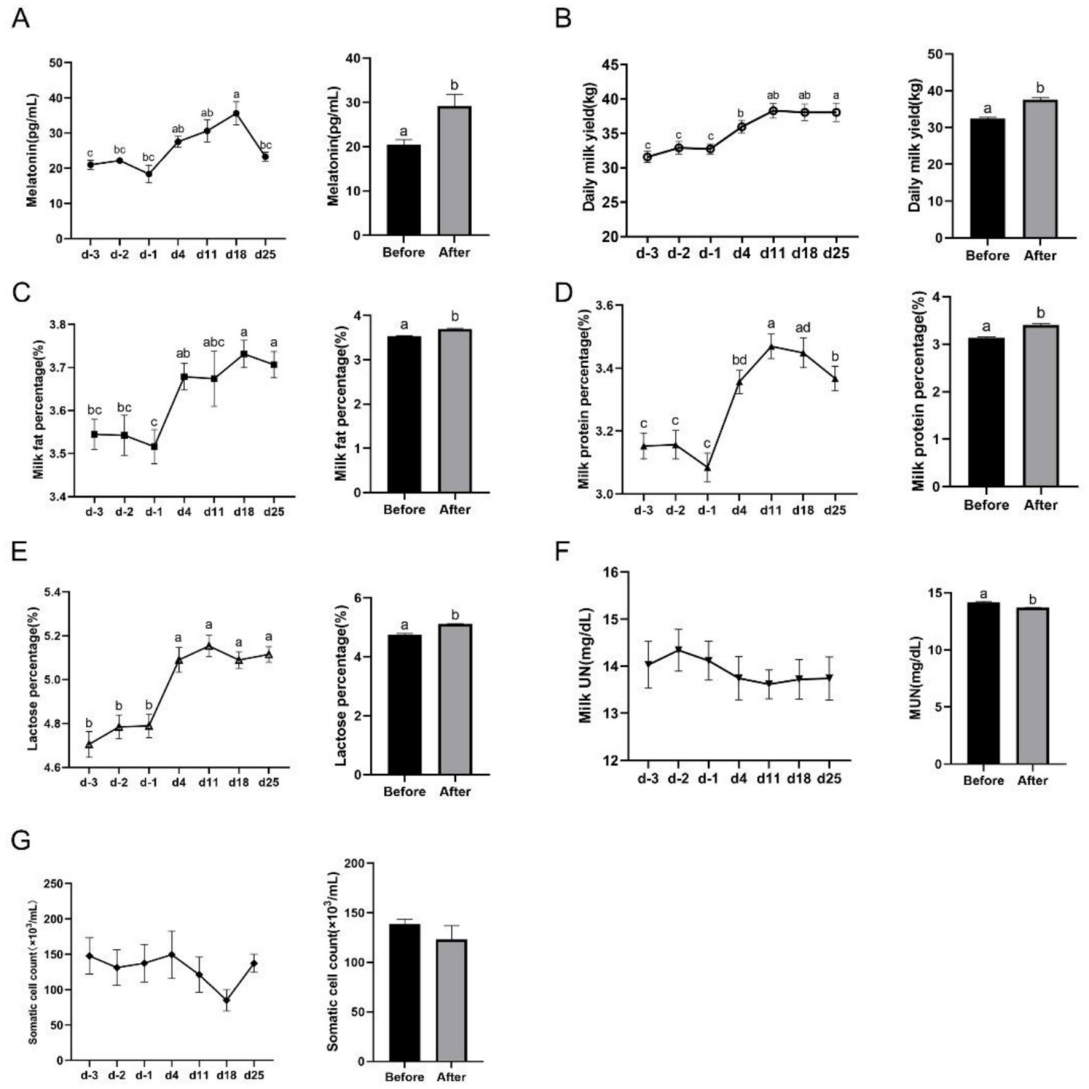
Effects of yellow light on milk quality and daily milk yield. **(A)** Milk MT content at different time points and the average content, *n* = 29; **(B)** daily milk yield at different time points and the average level, *n* = 18; **(C)** milk fat percentage at different time points and the average level, *n* = 29; **(D)** milk protein percentage at different time points and the average level, *n* = 29; **(E)** milk lactose percentage at different time points and the average level, *n* = 29; **(F)** milk UN content, *n* = 21; and **(G)** somatic cell count of the milk at different time points and the average level, *n* = 26. The data were expressed as mean ± SEM. Different letters indicated *P* < 0.05. The horizontal coordinate d-3, d-2, and d-1 represent the third, second, and the first days before yellow light exposure, while d4, d11, d18, and d25 represent the 4th, 11th, 18th, and 25th after yellow light exposure.

To confirm these observations, these parameters were compared in the same group before and after yellow light treatment. The results showed that the milk MT content was significantly higher after the yellow light exposure than that before the exposure (20.49–29.22 pg/ml; Figure 2A). The similar results was observed in the daily milk yield (32.39–37.58 kg; Figure 2B), the milk fat percentage (3.53%−3.70%; Figure 2C), the milk protein percentage (3.13%−3.41%; Figure 2D) and the lactose percentage (4.76%−5.11%; Figure 2E), While the milk UN content was significantly reduced after yellow light exposure compared to the values before the exposure (14.16–13.70 mg/dl; Figure 2F), and SCC in the milk were also reduced (138.60–123.00 × 10^3^/ml), but the reduction of SCC did not reach significant difference compared before the exposure (Figure 2G).

### 3.3 The potential association between milk MT content and the levels of milk protein, lactose, milk UN, and SCC

**Figure 3:**
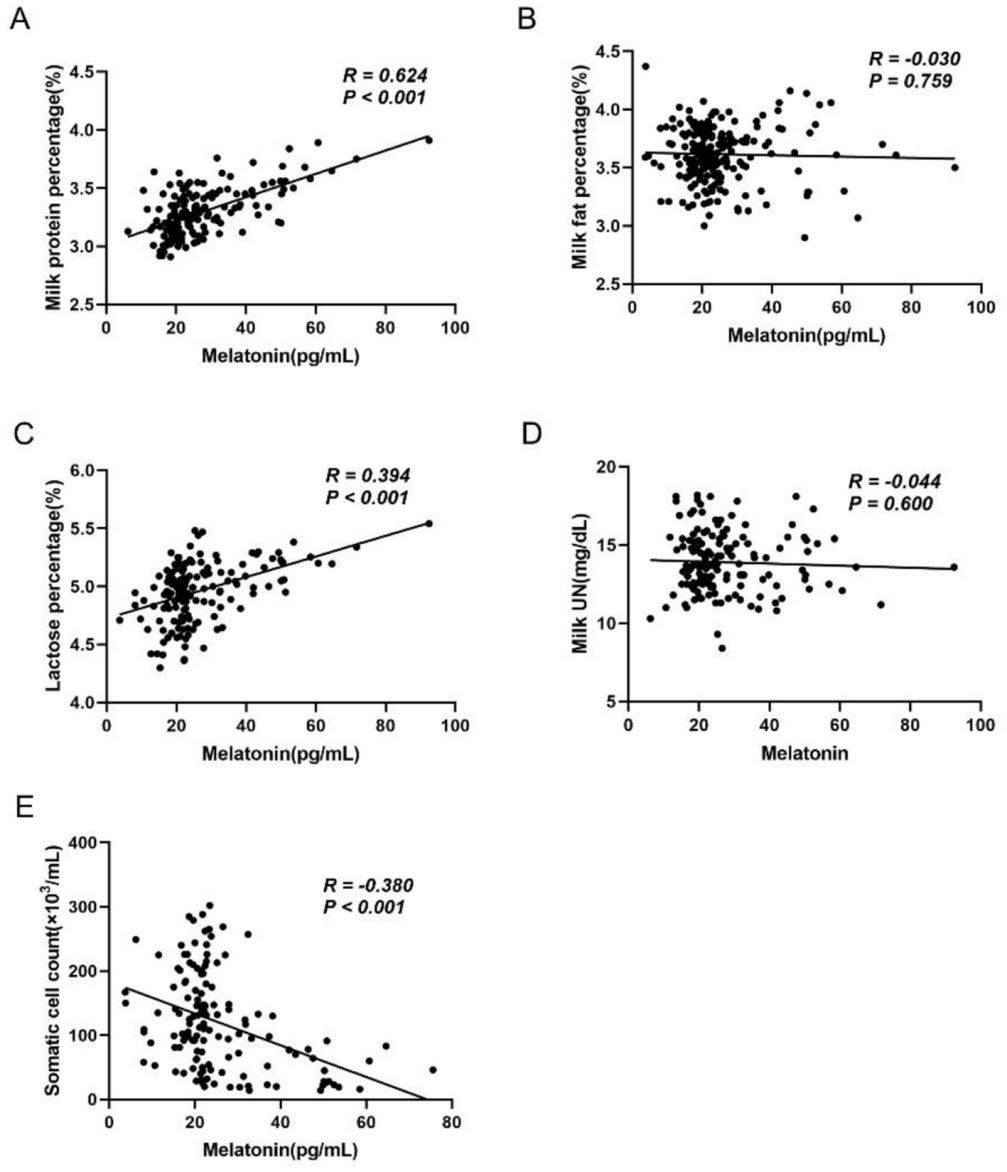
The potential association between milk MT content and milk composition. **(A)** Correlation analysis between MT and milk protein percentage, *n* = 149; **(B)** correlation analysis between MT and milk fat percentage, *n* = 219; **(C)** correlation analysis between MT and lactose percentage, *n* = 159; **(D)** correlation analysis between MT and milk UN, *n* = 147; and **(E)** correlation analysis between MT and somatic cell count, *n* = 136.

To explore the relationship between MT and milk quality, we conducted correlation analyses between these indicators and MT levels. The results from the linear correlation analysis showed a significantly positive correlation between milk MT content and milk protein percentage (*R* = 0.624, *P* < 0.001) and lactose percentage (*R* = 0.394, *P* < 0.001; Figures 3A, C). In contrast, a highly significant negative correlation was observed between milk MT content and milk SCC (*R* = −0.380, *P* < 0.001; Figure 3E). No significant correlation was found between milk MT content and milk fat percentage (*R* = −0.030, *P* > 0.05; Figure 3B) and milk UN (*R* = −0.044, *P* > 0.05; Figure 3D).

### 3.4 Effects of yellow light on serum MT levels and antioxidant capacity

**Figure 4:**
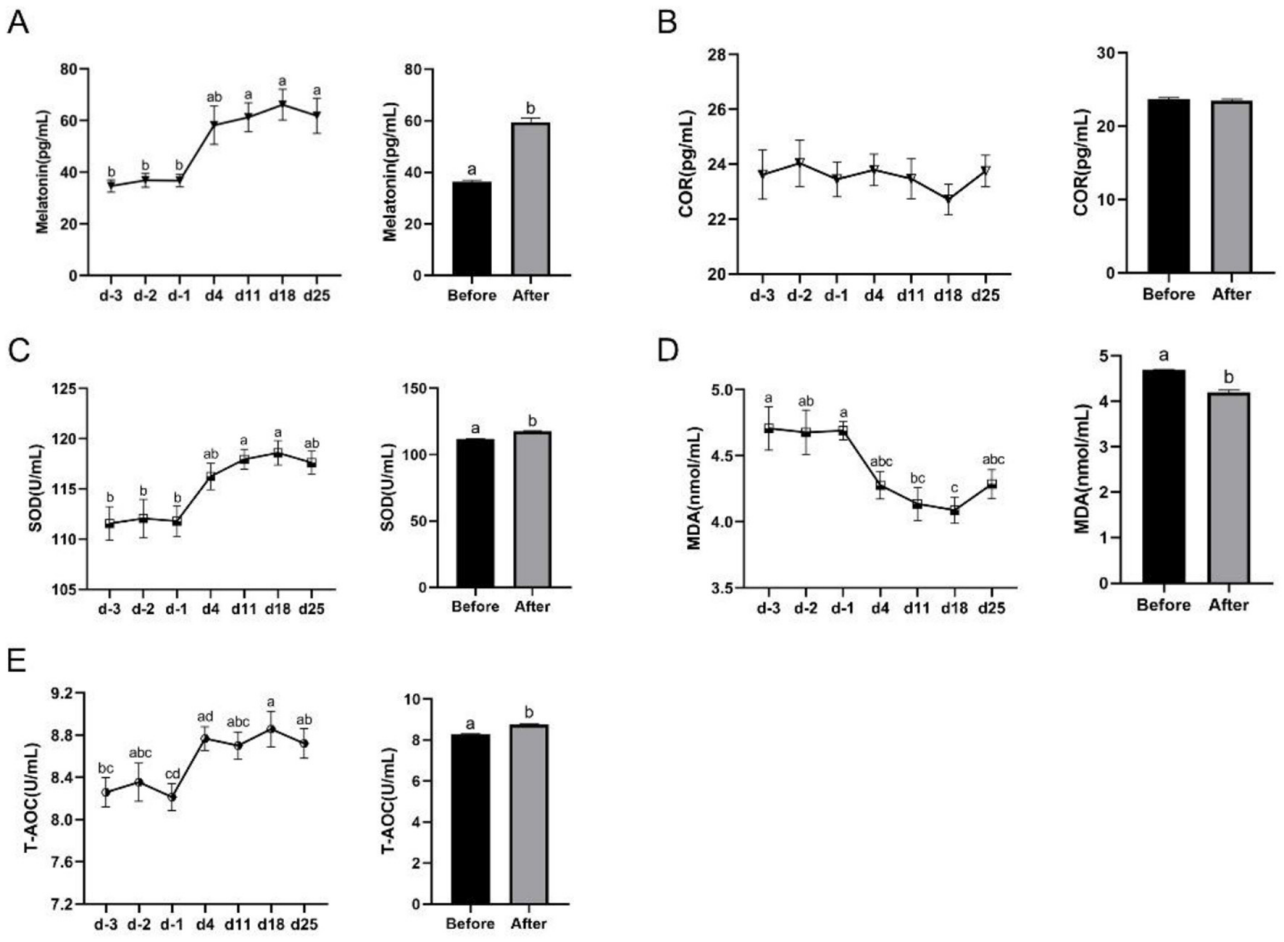
Effects of yellow light exposure on serum MT content and antioxidant indicators of cows. **(A)** Serum MT content at different time points and the average content, *n* = 29. **(B)** Serum COR content at different time points and the average level, *n* = 14; **(C)** serum SOD content at different time points and the average level, *n* = 14; **(D)** serum MDA content at different time points and the average level, *n* = 12; and **(E)** serum T-AOC content at different time points and the average level, *n* = 12. The data were expressed as mean ± SEM. Different letters indicated *P* < 0.05. The horizontal coordinate d-3, d-2, and d-1 represent the third, second and the first days before yellow light exposure, while d4, d11, d18, and d25 represent the 4th, 11th, 18th and 25th after yellow light exposure.

We then examined the effect of yellow light on serum MT levels and antioxidant capacity in dairy cows by evaluating relevant indicators. The results showed that the overall level of serum MT in cows after yellow light exposure was significantly higher than that before their exposure (36.30–59.48 pg/ml; Figure 4A), but the yellow light exposure had little effects on the serum COR content compared to the level before exposure (Figure 4B). The yellow light exposure significantly elevated the serum SOD (111.80–117.60 U/ml; Figure 4C) and T-AOC (8.28–8.76 U/ml; Figure 4E) levels, but significantly lowered the serum MDA level (4.69–4.20 nmol/ml; Figure 4D).

### 3.5 Effects of yellow light exposure on the levels of proinflammatory cytokines

**Figure 5:**
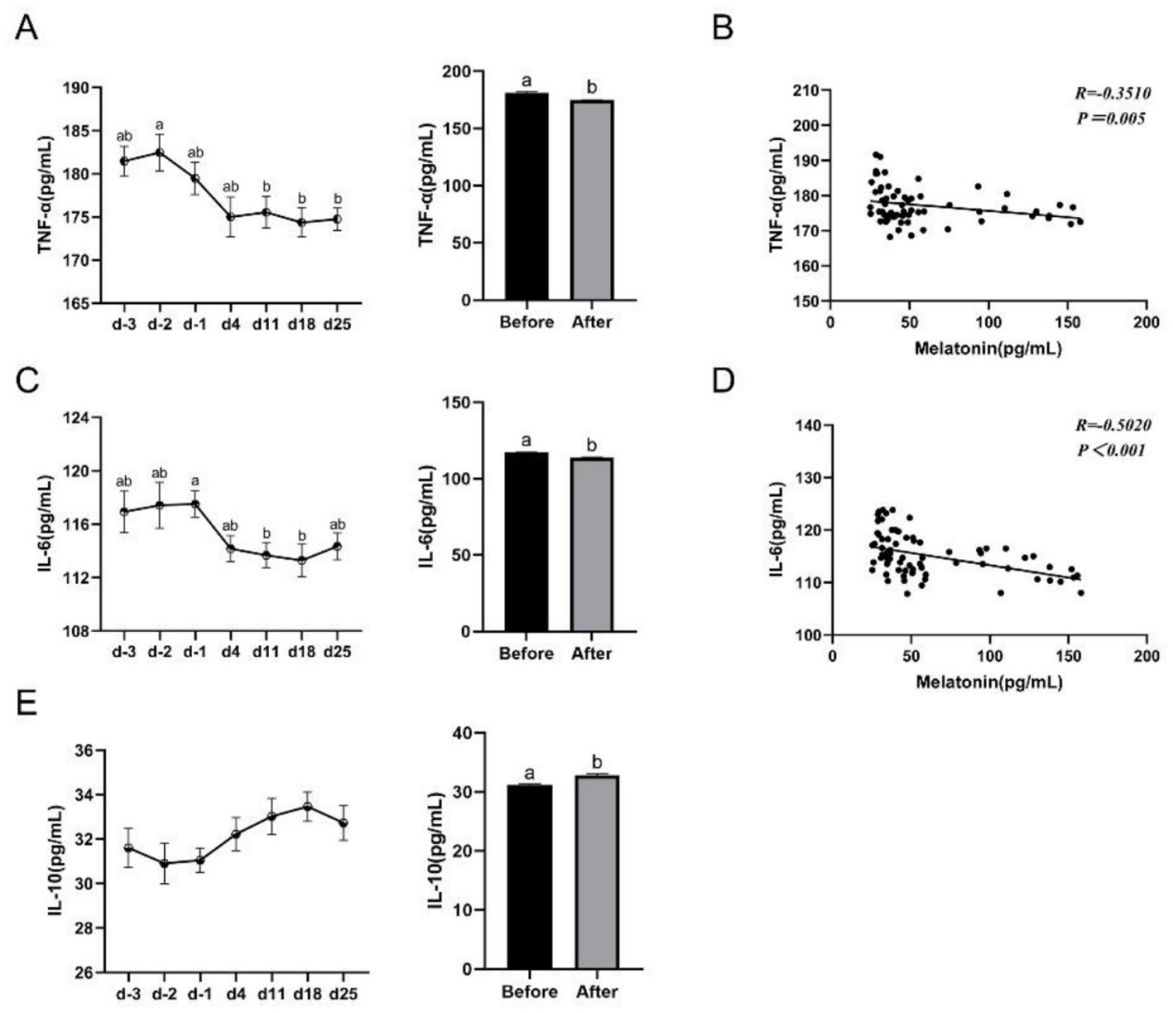
Effects of yellow light on the proinflammatory cytokines of dairy cows. **(A)** Serum TNF-α content at different time points and the average level, *n* = 11; **(B)** correlation analysis between MT and TNF-α, *n* = 62; **(C)** serum IL-6 content at different time points and the average level, *n* = 12; **(D)** correlation analysis between MT and IL-6, *n* = 75; and **(E)** serum IL-10 content at different time points and the average level, *n* = 12. The data were expressed as mean ± SEM. Different letters indicated *P* < 0.05. The horizontal coordinate d-3, d-2, and d-1 represent the third, second and the first days before yellow light exposure, while d4, d11, d18, and d25 represent the 4th, 11th, 18th, and 25th after yellow light exposure.

To assess changes in immune function, we measured representative indicators: TNF-α, IL-6, and IL-10. Correlation analyses were then performed between MT and each of TNF-α and IL-6. The results showed that yellow light exposure significantly lowered the serum TNF-α (181.10–174.90 pg/ml; Figure 5A) and IL-6 levels (117.30–113.90 pg/ml; Figure 5C) compared to the values before exposure. However, yellow light exposure significantly increased the serum IL-10 level compared to the value before exposure (31.18–32.86 pg/ml; Figure 5E). The results of the linear correlation analysis showed a significantly negative correlation between serum MT and TNF-α (*R* = −0.3510, *P* = 0.005; Figure 5B) as well as IL-6 levels (*R* = −0.5020, *P* < 0.001; Figure 5D).

### 3.6 Effects of yellow light exposure on reproductive hormones and performance

**Figure 6:**
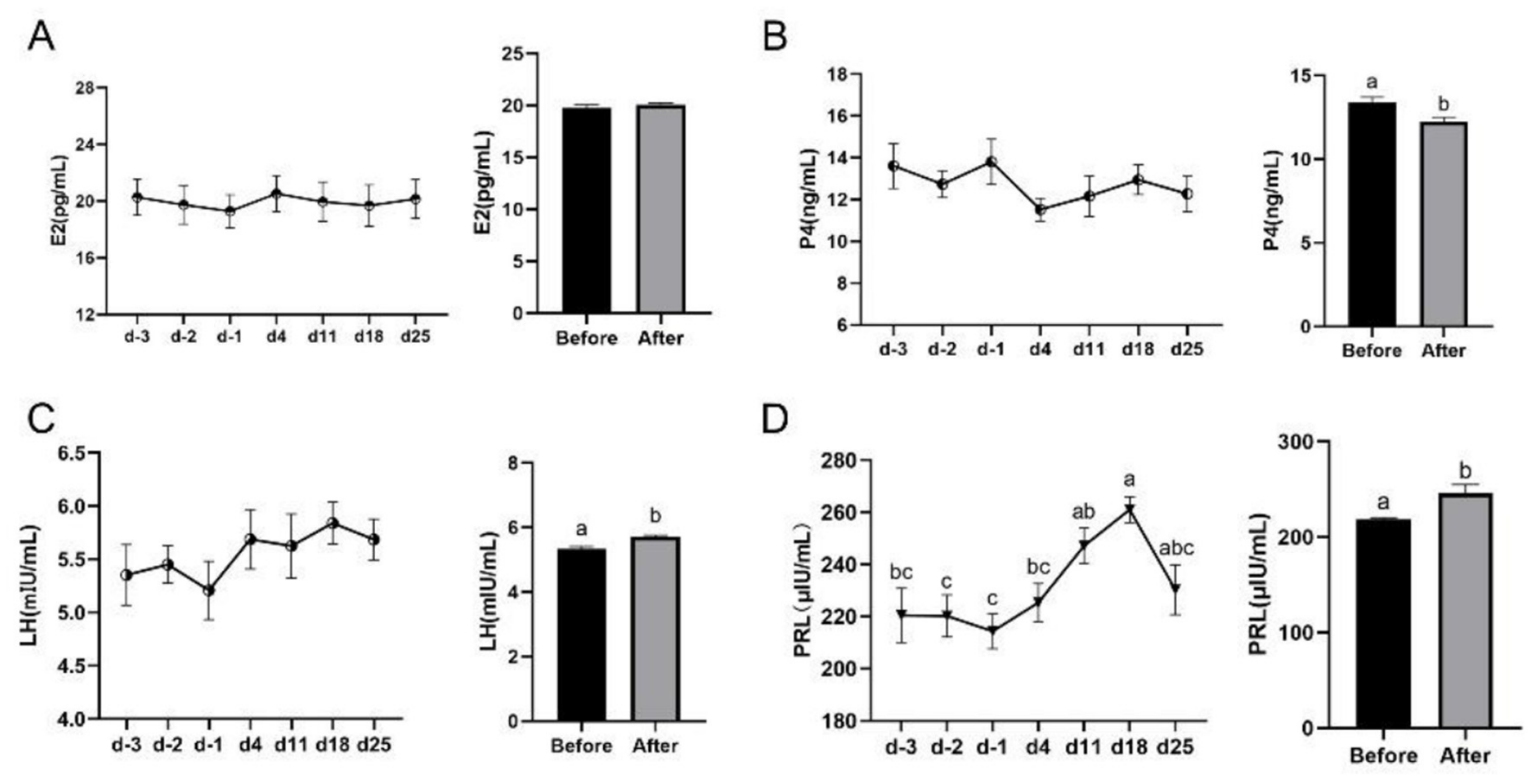
Effects of yellow light exposure on reproduction hormones of cows. **(A)** Serum E2 content at different time points and the average level, *n* = 10; **(B)** serum P4 content at different time points and the average level, *n* = 10; **(C)** serum LH content at different time points and the average level, *n* = 13; and **(D)** serum PRL content at different time points and the average level, *n* = 14. The data were expressed as mean ± SEM. Different letters indicated *P* < 0.05. The horizontal coordinate d-3, d-2, and d-1 represent the third, second and the first days before yellow light exposure, while d4, d11, d18, and d25 represent the 4th, 11th, 18th, and 25th after yellow light exposure.

To investigate the effects of yellow light treatment on reproductive hormones and performance, we analyzed the changes in E2, P4, LH, and PRL levels before and after the treatment. We also tracked the reproductive metrics of cows exposed to yellow light compared to those not exposed. The results showed that there were significant differences in the levels of LH (5.336–5.708 mIU/ml) and PRL (218.4–246.1 μIU/ml) after yellow light exposure (Figures 6C, D). However, the yellow light exposure significantly decreased the P4 level compared to the value before the exposure (Figure 6B). There was no significant difference in the E2 level (Figure 6A). Compared to the control group, cows exposed to yellow light showed significant improvements in reproductive metrics (Table 1): the interval to first postpartum estrous cycle was shortened (61.72 ± 1.27 to 56.91 ± 1.14 days), the pregnancy rate after first-insemination was increased (23.68 ± 4.42% to 38.15 ± 5.00%), and the pregnancy days after first-insemination were reduced (96.84 ± 4.88 to 82.95 ± 4.50 days).

**Table 1:** Effects of yellow light exposure on reproductive performance of cows.

**Group**	**No. of dairy cows**	**Postpartum interval (d)**	**No. of pregnant cows**	**Pregnancy rate (%)**	**Pregnancy days (d)**
Normal	76	61.72 ± 1.27^a^	18	23.68 ± 4.42^b^	96.84 ± 4.88^a^
Yellow light	76	56.91 ± 1.14^b^	29	38.15 ± 5.00^a^	82.95 ± 4.50^b^

Postpartum interval: the interval to first postpartum estrous cycle. Pregnancy rate: the pregnancy rate after first-insemination. Pregnancy days: the pregnancy days after first-insemination. The data were expressed as mean ± SEM. Different letters indicated *P* < 0.05.

## 4 Discussion

Light color and photoperiod significantly affect the life activities of animals (15, 16). In this study, we systematically investigated the effects of light with different wavelengths on dairy cows through two experiments, with a particular focus on the impact of yellow light on production performance, milk composition, and health status. Our results demonstrated that yellow light treatment significantly improved the immune function, antioxidant capacity, production performance, and reproductive performance of dairy cows. The key factor triggering this series of responses may be the significant increase in MT levels in both serum and milk of the cows. These findings collectively indicate that yellow light exposure, as a non-invasive management strategy, has great potential to enhance both economic returns and animal welfare on dairy farms through the regulation of endogenous MT.

### 4.1 Yellow light alleviates melatonin inhibition and enhances its synthesis

As early as 2006, Gnann proposed a method for producing high-MT milk, which involved initially using blue-enriched light to inhibit MT secretion, followed by exposure to at least one light source above 500 nm (capable of emitting red, orange, yellow, or mixed light) for 2 h to maximally reverse MT suppression, thereby yielding dairy products with elevated MT levels. Our findings are consistent with this approach, demonstrating that yellow light positively influences MT concentrations in milk. Moreover, we observed that yellow light was more effective than red light in enhancing MT levels in both milk and serum. These results are consistent with another study, which reported that yellow LED light increased serum MT levels in dairy cows compared to blue LED light (9). The effects rely on the melatonin synthesis mechanism in the pineal gland. In mammals, light information is detected by intrinsically photosensitive retinal ganglion cells (ipRGCs). This signal is transmitted via the retinohypothalamic tract (RHT) to the suprachiasmatic nucleus (SCN), which houses the circadian pacemaker (17). Through a polysynaptic neural pathway, the SCN controls the release of norepinephrine from sympathetic nerve endings in the pineal gland. This results in a rapid increase in the activity and expression of the melatonin-synthesizing enzyme AANAT, thereby promoting melatonin synthesis and secretion (18, 19).

Photoreceptors exhibit varying sensitivities to different light wavelengths. Specifically, melanopsin-containing ipRGCs function as non-visual photoreceptors (20). They show highest sensitivity to short-wavelength light (e.g., blue light at 480 nm) and lowest sensitivity to red light (18). Short-wavelength light signals (indicating daytime) received by melanopsin travel along the RHT to the SCN. The SCN releases GABA, which disinhibits the paraventricular nucleus (PVN) of the hypothalamus. This action suppresses the sympathetic pathway to the pineal gland, ultimately inhibiting melatonin synthesis. At night, the SCN ceases GABA production, lifting the inhibition on the downstream pathway (19). In our results, melatonin levels in the yellow light treatment group were markedly higher than in the dark control group. This suggests that yellow light may affect the pathway through multiple mechanisms. Firstly, it could suppress GABA synthesis, removing the inhibition on melatonin production. Secondly, it might enhance norepinephrine release from pineal sympathetic nerves, further stimulating melatonin synthesis.

### 4.2 Yellow light improves milk quality via melatonin

Subsequent analysis demonstrated that yellow light treatment significantly enhanced milk yield, milk fat percentage, milk protein percentage, and lactose content, whereas milk UN decreased significantly and SCC exhibited a decreasing trend. Further correlation analysis indicated that changes in milk protein percentage, lactose content, and SCC were significantly associated with MT levels. Numerous studies support the beneficial role of MT in enhancing milk quality. For instance, Li et al. (21) reported that dietary supplementation with 120 mg of MT for 30 days increased milk fat and protein percentages while reducing SCC. Similarly, Wu et al. (22) demonstrated that exogenous MT administration improved Dairy Herd Improvement (DHI) parameters. Milk UN, as another important component in milk, reflects the balance of dietary nutrition and is commonly used to evaluate protein utilization efficiency. High milk UN levels indicate excessive dietary crude protein or insufficient non-structural carbohydrates in the diet, while low milk UN suggests a need to reassess sources of dietary protein and carbohydrates. Multiple studies have shown that dairy cow lameness (23), ketosis (24), and abnormal reproductive performance (25) are associated with milk UN levels. During our trial, although the structure of the dairy cows' diet remained unchanged, the decrease in milk UN and increase in milk protein indicate that yellow light improved the efficiency of protein utilization, which positively influenced the health status and reproductive performance of the cows.

In the present study, we also observed an increase in serum prolactin levels following yellow light treatment, which may directly contribute to the elevated milk yield, given the established lactogenic role of prolactin in dairy cattle (26). The secretion of prolactin may be simultaneously regulated by light and melatonin. Melatonin stimulates PRL expression and secretion by promoting the Nrg1/ErbB4 signaling pathway (27). However, previous research reported that exogenous melatonin inhibits prolactin secretion in mammary epithelial cells of dairy goats (28), and we speculate that this opposite effect is dose-dependent. Similar to melatonin, prolactin exhibits a circadian rhythm, increasing in darkness and decreasing under light exposure (29). In this study, we propose that yellow light directly elevates serum melatonin and prolactin levels, but an interaction between melatonin and prolactin is likely. In summary, using yellow light at night can both increase milk yield and improve milk composition, thereby comprehensively enhancing dairy cow milk performance.

### 4.3 Melatonin induced by yellow light mediates antioxidant and anti-inflammatory responses

Oxidative stress and inflammation influence each other (30). Oxidative phenomena can transmit and manage signals during the initial stages of inflammation (31). Meanwhile, the secretion of substantial reactive substances by inflammatory cells contributes to increased oxidative stress at the site of inflammation.

In this study, with the accumulation of days under yellow light treatment, the level of IL-10 in dairy cow serum gradually increased, while the levels of TNF-α and IL-6 progressively decreased. In addition, the MDA level was lower than the pre-treatment level, and the levels of SOD and T-AOC were elevated. These findings suggest that both immune and antioxidant capacities were improved after yellow light treatment. Research shows that the recovery rate of cow mastitis treated with antibiotics combined with low-intensity laser irradiation at 870–970 nm was significantly higher than that of the antibiotic-only treatment group, indicating that light can influence the body's inflammatory response (32). Patients with melasma treated with topical depigmenting serum combined with 577 nm yellow light laser had significantly higher serum T-AOC and SOD1 levels than those treated with topical depigmenting serum alone (33). These are consistent with our conclusion that yellow light can enhance the body's antioxidant and anti-inflammatory capacity. Subsequently, we found that serum MT was significantly negatively correlated with TNF-α and IL-6. MT is an effective antioxidant and anti-inflammatory agent (34). Therefore, we speculate that these changes induced by yellow light are closely related to increased MT levels in the body. Yu et al. (35) confirmed that MT exerts both antioxidant and anti-inflammatory effects on bovine mammary epithelial cells: it reduces TNF-α and IL-6 mRNA expression in LPS-stimulated cells via the TLR4 signaling pathway, thereby decreasing inflammatory responses; it also activates the Nrf2 antioxidant defense pathway to inhibit oxidative stress. Li et al. (21) fed mid-to-late lactation cows 120 mg of MT for 30 days and found significantly reduced serum TNF-α, IL-6 and MDA levels, while improved serum IL-10 and SOD levels. In this study, yellow light treatment increased melatonin levels, and melatonin exerts antioxidant effects through multiple pathways. On one hand, melatonin and its metabolites directly bind to free radicals, forming a cascade reaction that efficiently scavenges reactive oxygen species. On the other hand, melatonin activates the antioxidant defense system SOD, ultimately increasing T-AOC and decreasing MDA. In previous studies, melatonin has been shown to mediate anti-inflammatory effects via the nuclear factor kappaB pathway (36), TLR4 pathway (35), and Nrf2 pathway (34). Here, we only detected changes in inflammatory factors, and more specific mechanisms require further investigation.

### 4.4 Improved reproductive performance with enhanced general health

In this study, yellow light treatment resulted in decreased serum P4 levels and increased PRL and LH levels in dairy cows, suggesting that the cows were in the transition phase from the luteal phase to the follicular phase. Combined with the shortened the interval to first postpartum estrous cycle, increased pregnancy rate, and reduced pregnancy days, we speculate that yellow light treatment enabled cows to recover more rapidly from the pregnancy-lactation physiological state, initiate normal ovarian cyclicity, and successfully complete subsequent pregnancy. We propose that these improvements in reproductive indices depend on the enhanced overall health status of cows after yellow light treatment, including improved protein utilization, immune function, and antioxidant capacity. This is because studies have shown that inflammation, oxidative stress, and malnutrition can adversely affect reproductive performance in dairy cows. For example, mastitis affects not only the udder but also extends to the entire reproductive system, leading to decreased conception rates, abnormal estrous cycles, early embryonic death or abortion, as well as increased days open and service index (number of artificial insemination (AI) required per conception) and reduced conception rates (37). Kumar et al. (37) speculated that this might be related to factors such as mastitis pathogens, infection duration, and excessive cytokine production during the disease process. Japanese Black cattle with poor reproductive performance exhibited significantly higher oxidative stress indices in uterine fluid, while the concentration of antioxidants in the uterus positively influenced the uterine environment and subsequent establishment of successful pregnancy in Japanese Black cattle (38). During pregnancy in dairy cows, the antioxidant status of uterine luminal cells, peripheral blood MT concentration, and local expression of antioxidant cell markers all influence embryo quality and pregnancy success (39). Studies have shown that both excessively high and low MUN concentrations are detrimental to dairy cow reproduction, with varying thresholds among breeds (40).

## 5 Conclusions

**Figure 7:**
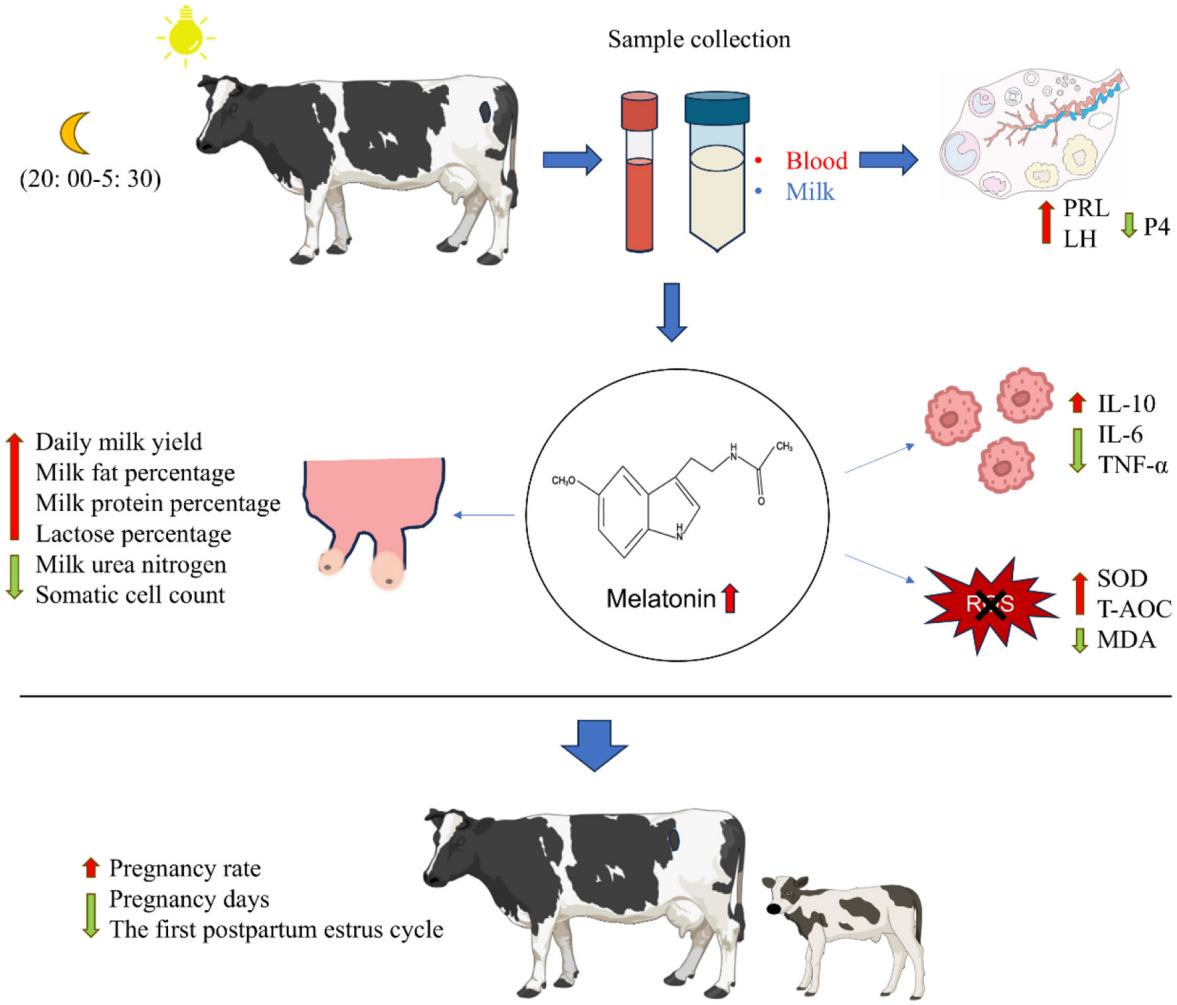
The potential activities of yellow light at night on the cows.

Overall, our study demonstrates that yellow light increases MT levels in both milk and serum, and MT subsequently influences dairy productivity, immune function, antioxidant status, and reproductive performance (Figure 7). These findings support the implementation of yellow light exposure at night as a feasible lighting management strategy in practical production. Currently, we are trying to further refine the feasibility of this light technology through experiments. We are applying it to different species and reducing light duration and intensity, aiming to reduce farm input costs and increase returns. Meanwhile, we attempt to further explore the underlying mechanisms of how yellow light precisely influences MT. We also want to know the specific mechanisms by which MT modulates cytokines and oxidative markers. Thereby addressing the limitations of this study.

## Data Availability

The raw data supporting the conclusions of this article will be made available by the authors, without undue reservation.

## References

[1] Dauchy RT Blask DE. Vivarium lighting as an important extrinsic factor influencing animal-based research. J Am Assoc Lab Anim Sci. (2023) 62:3–25. doi: 10.30802/AALAS-JAALAS-23-000003 36755210 PMC9936857

[2] Hodnik JJ Jankovec M Ježek J Krušič Ž Mitterhofer S Starič J. Effects of Uv-B light exposure during automatic milking on vitamin D levels in holstein friesian cows. Front Vet Sci. (2025) 11:1433230. doi: 10.3389/fvets.2024.1433230 39881717 PMC11776301

[3] Stahl TC Mullin EM Piñeiro JM Lunak M Chahine M Erickson PS. Creating models for the prediction of colostrum quantity, quality, and immunoglobulin g yield in multiparous jersey cows from performance in the previous lactation and environmental changes. J Dairy Sci. (2024) 107:4855–70. doi: 10.3168/jds.2023-24209 38278293

[4] Auchtung TL Salak-Johnson JL Morin DE Mallard CC Dahl GE. Effects of photoperiod during the dry period on cellular immune function of dairy cows. J Dairy Sci. (2004) 87:3683–9. doi: 10.3168/jds.S0022-0302(04)73507-9 15483152

[5] Wall EH Auchtung-Montgomery TL Dahl GE McFadden TB. Short communication: short-day photoperiod during the dry period decreases expression of suppressors of cytokine signaling in mammary gland of dairy cows. J Dairy Sci. (2005) 88:3145–8. doi: 10.3168/jds.S0022-0302(05)72997-0 16107404

[6] Alward KJ Duncan AJ Ealy AD Dahl GE Petersson-Wolfe CS Cockrum RR. Changes in photoperiod during the dry period impact colostrum production in Holstein and Jersey cows. J Dairy Sci. (2025) 108:1672–85. doi: 10.3168/jds.2024-25415 39662799

[7] Lim D-H Kim T-I Park S-M Ki K-S Kim Y. Effects of photoperiod and light intensity on milk production and milk composition of dairy cows in automatic milking system. J Anim Sci Technol. (2021) 63:626–39. doi: 10.5187/jast.2021.e59 34189510 PMC8204001

[8] Palubinskas G Žilaitis V Antanaitis R. Improvement of dairy cow embryo yield with low level laser irradiation. Pol J Vet Sci. (2017) 20:307–12. doi: 10.1515/pjvs-2017-0037 28865223

[9] Elsabagh M Mon M Takao Y Shinoda A Watanabe T Kushibiki S . Exposure to blue led light before the onset of darkness under a long-day photoperiod alters melatonin secretion, feeding behaviour and growth in female dairy calves. Anim Sci J. (2020) 91:e13353. doi: 10.1111/asj.13353 32219969

[10] Baiocchi L Zhou T Liangpunsakul S Ilaria L Milana M Meng F . Possible application of melatonin treatment in human diseases of the biliary tract. Am J Physiol Gastrointest Liver Physiol. (2019) 317:G651–60. doi: 10.1152/ajpgi.00110.2019 31509434 PMC6879895

[11] Labani N Gbahou F Noblet M Masri B Broussaud O Liu J . Pistacia Vera extract potentiates the effect of melatonin on human melatonin Mt1 and Mt2 receptors with functional selectivity. Pharmaceutics. (2023) 15:1845. doi: 10.3390/pharmaceutics15071845 37514032 PMC10386454

[12] Mayo JC Sainz RM González-Menéndez P Hevia D Cernuda-Cernuda R. Melatonin transport into mitochondria. Cell Mol Life Sci. (2017) 74:3927–40. doi: 10.1007/s00018-017-2616-8 28828619 PMC11107582

[13] Boulanger V Zhao X Lacasse P. Protective effect of melatonin and catalase in bovine neutrophil-induced model of mammary cell damage1. J Dairy Sci. (2002) 85:562–9. doi: 10.3168/jds.S0022-0302(02)74109-X 11949860

[14] Touitou Y Reinberg A Touitou D. Association between light at night, melatonin secretion, sleep deprivation, and the internal clock: health impacts and mechanisms of circadian disruption. Life Sci. (2017) 173:94–106. doi: 10.1016/j.lfs.2017.02.008 28214594

[15] Inabu Y Takakura Y Shinohara Y Sunadome M Watanabe R Kushibiki S . Comparison of milk production and endocrine profiles of dairy cows exposed to either white light-emitting diode or induction lighting. Domest Anim Endocrinol. (2025) 93:106958. doi: 10.1016/j.domaniend.2025.106958 40516157

[16] Li C Shu H Gu X. Photoperiod management in farm animal husbandry: a review. Animals. (2025) 15:591. doi: 10.3390/ani15040591 40003072 PMC11851680

[17] Jiang N Wang Z Cao J Dong Y Chen Y. Role of Monochromatic light on daily variation of clock gene expression in the pineal gland of chick. J Photochem Photobiol B: Biol. (2016) 164:57–64. doi: 10.1016/j.jphotobiol.2016.09.020 27643985

[18] Lewczuk B Martyniuk K Szyryńska N Prusik M Ziółkowska N. Monochromatic photophase light alters diurnal profiles of melatonin pathway indoles in the Rat Pineal gland. Int J Mol Sci. (2025) 13:6515. doi: 10.20944/preprints202505.2278.v1 40650290 PMC12250469

[19] Pevet P Challet E. Melatonin: both master clock output and internal time-giver in the circadian clocks network. J Physiol-Paris. (2011) 105:170–82. doi: 10.1016/j.jphysparis.2011.07.001 21914478

[20] Pilorz V Tam SKE Hughes S Pothecary CA Jagannath A Hankins MW . Melanopsin regulates both sleep-promoting and arousal-promoting responses to light. PLoS Biol. (2016) 14:e1002482. doi: 10.1371/journal.pbio.1002482 27276063 PMC4898879

[21] Li Y Cheng Z Ma W Qiu Y Liu T Nan B . Effect of exogenous melatonin on performance and mastitis in dairy cows. Vet Sci. (2024) 11:431. doi: 10.3390/vetsci11090431 39330810 PMC11435509

[22] Wu H Yao S Wang T Wang J Ren K Yang H . Effects of melatonin on dairy herd improvement (DHI) of holstein cow with high SCS. Molecules. (2021) 26:834. doi: 10.3390/molecules26040834 33562613 PMC7915447

[23] Necula D-C Warren HE Taylor-Pickard J Simiz E Stef L. Associations of lameness with indicators of nitrogen metabolism and excretion in dairy cows. Agriculture. (2022) 12:2109. doi: 10.3390/agriculture12122109

[24] Rius AG McGilliard ML Umberger CA Hanigan MD. Interactions of energy and predicted metabolizable protein in determining nitrogen efficiency in the lactating dairy cow. J Dairy Sci. (2010) 93:2034–43. doi: 10.3168/jds.2008-1777 20412918

[25] Webb EC de Bruyn E. Effects of milk urea nitrogen (MUN) and climatological factors on reproduction efficiency of holstein friesian and jersey cows in the subtropics. Animals. (2021) 11:3068. doi: 10.3390/ani11113068 34827800 PMC8614443

[26] Yu M Feng B Bean JC Zhao Q Yang Y Liu H . Suppression of hypothalamic oestrogenic signal sustains hyperprolactinemia and metabolic adaptation in lactating mice. Nat Metab. (2025) 7:759–77. doi: 10.1038/s42255-025-01268-z 40211044 PMC12509271

[27] Zhang W. Dao J-j, Li Q, Liu C, Qiao C-m, Cui C, et al. Neuregulin 1 Mitigated prolactin deficiency through enhancing trpm8 signaling under the influence of melatonin in senescent pituitary lactotrophs. Int J Biol Macromol. (2024) 275:133659. doi: 10.1016/j.ijbiomac.2024.133659 38969045

[28] Zhang W Chen J Zhao Y Zheng Z Song Y Wang H . The inhibitory effect of melatonin on mammary function of lactating dairy goats. Biol Reprod. (2018) 100:455–67. doi: 10.1093/biolre/ioy223 30346485

[29] Tóth A Dobolyi Á. Prolactin in sleep and eeg regulation: new mechanisms and sleep-related brain targets complement classical data. Neurosci Biobehav Rev. (2025) 169:106000. doi: 10.1016/j.neubiorev.2024.106000 39755290

[30] Bellanti F Coda ARD Trecca MI Lo Buglio A Serviddio G Vendemiale G. Redox imbalance in inflammation: the interplay of oxidative and reductive stress. Antioxidants. (2025) 14:656. doi: 10.3390/antiox14060656 40563291 PMC12189482

[31] Radogna F Diederich M Ghibelli L. Melatonin: a pleiotropic molecule regulating inflammation. Biochem Pharmacol. (2010) 80:1844–52. doi: 10.1016/j.bcp.2010.07.041 20696138

[32] Malinowski E Krumrych W Markiewicz H. The effect of low intensity laser irradiation of inflamed udders on the efficacy of antibiotic treatment of clinical mastitis in dairy cows. Vet Ital. (2019) 55:253–60. 31599550 10.12834/VetIt.818.3989.2

[33] Khurana N Mehdi MM Sawhney MPS Bansal SK. Assessment of systemic oxidative stress modulation in melasma following treatment with yellow light laser 577 Nm and topical 0.3% 4n butyl resorcinol: a comparative prospective study. Indian J Dermatol. (2025) 70:57–62. doi: 10.4103/ijd.ijd_11_24 40162352 PMC11952710

[34] Lim H-S Park J Whang W-J Kang WS Kim S Yoo Y-S . Melatonin ameliorates desiccation stress-induced ocular inflammation in an *in vitro* model by activating the Nrf2 pathway. J Cell Mol Med. (2025) 29:e70879. doi: 10.1111/jcmm.70879 41102912 PMC12530958

[35] Yu G-M Kubota H Okita M Maeda T. The anti-inflammatory and antioxidant effects of melatonin on lps-stimulated bovine mammary epithelial cells. PLoS ONE. (2017) 12:e0178525. doi: 10.1371/journal.pone.0178525 28542575 PMC5444821

[36] Li W Chen Q Fu H. The role of melatonin in affecting cognitive dysfunction in acute sleep deprivation mice through the nuclear factor kappab pathway and oxidative stress. Transl Neurosci. (2025) 16:20250379. doi: 10.1515/tnsci-2025-0379 41079404 PMC12514685

[37] Kumar N Manimaran A Kumaresan A Jeyakumar S Sreela L Mooventhan P . Mastitis effects on reproductive performance in dairy cattle: a review. Trop Anim Health Prod. (2017) 49:663–73. doi: 10.1007/s11250-017-1253-4 28283873

[38] Hagita Y Miura R Shirasuna K Ajito T Matsumoto H. Effects of oxidative stress and antioxidant activity in plasma and uterine fluid during early postpartum on subsequent reproductive performance of Japanese black cows. Animals. (2025) 15:767. doi: 10.3390/ani15060767 40150296 PMC11939288

[39] Dirandeh E Ansari-Pirsaraei Z Thatcher W. Melatonin as a smart protector of pregnancy in dairy cows. Antioxidants. (2022) 11:292. doi: 10.3390/antiox11020292 35204175 PMC8868556

[40] Zhao X Zheng N Zhang Y Wang J. The role of milk urea nitrogen in nutritional assessment and its relationship with phenotype of dairy cows: a review. Anim Nutr. (2025) 20:33–41. doi: 10.1016/j.aninu.2024.08.007 39949732 PMC11821394

