# Supplementary figures and images for: Yellow light improves milk quality, antioxidant capacity, immunity, and reproductive ability in dairy cows by elevating endogenous melatonin
Source: Front Vet Sci. 2026 Jan 16;12:1730661. doi: 10.3389/fvets.2025.1730661 (PMC12856941; doi:10.3389/fvets.2025.1730661)

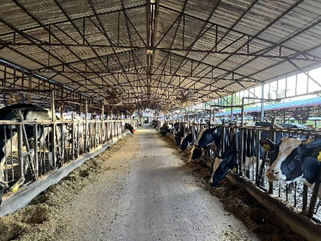

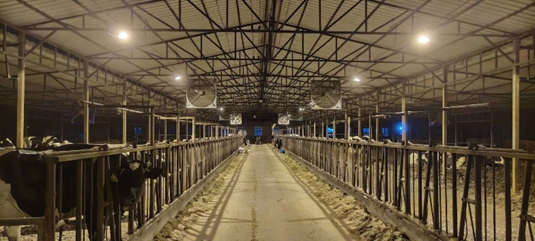


Supplementary Figure 1. Semi-open barn

Supplement: Supplementary file 1 [file Supplementary_file_1.doc]
